# Analysis of the efficacy of holmium laser and pneumatic ballistic in the treatment of impacted ureteral calculi

**DOI:** 10.1097/MD.0000000000021692

**Published:** 2020-09-04

**Authors:** Yang Chunlin, Du Wanlin, Du Jinhua

**Affiliations:** Department of Urology, Yuechi People's Hospital, Guang’an, Sichuan, China.

**Keywords:** complications, holmium laser, impacted ureteral calculi, pneumatic ballistics, stone-free rate, ureteroscopic lithotripsy

## Abstract

To explore the safety and effectiveness of ureteroscopic holmium laser lithotripsy (UHLL) and ureteroscopic pneumatic lithotripsy (UPL) in the treatment of impacted ureteral calculi (IUC).

Clinical data of 280 patients in our hospital from April 2016 to May 2019 were retrospectively collected and analyzed, including 136 cases of UHLL group and 144 cases of UPL group. The general clinical data, operation time, intraoperative bleeding volume, hospital stay, stone-free rate (SFR), and surgical complications were collected and analyzed in 2 group.

Compared with UPL group, the operation time of UHLL group was significantly reduced (27.25 ± 8.39 vs 34.32 ± 10.57, *P* < .05), but the hospitalization cost was significantly increased (9.25 ± 0.75 vs 8.24 ± 0.51, *P* < .05). In terms of total SFR, the UHLL group was significantly higher than the UPL group (93.38% vs 83.33%, *P* = .011). For proximal IUC, compared with the UPL group, the SFR of the UHLL group was significantly increased (88.33% vs 70.31%, *P* = 0.005). For distal IUC, there was no significant difference in SFR (97.37% vs 93.75%, *P* = .638) between the UHLL group and UPL group. There were no significant differences in the complications of local mucosal injury, hematuria, febrile urinary tract infection, ureteral perforation, and urinary sepsis in the 2 groups (*P* > .05). However, the UHLL group was significantly lower in stone residual rate than the UPL group (6.61% vs 16.67%, *P* = .001).

This study found that UHLL and UPL are safe and effective in the treatment of IUC, but UHLL has the advantages of shorter operation time and high SFR in the treatment of IUC.

## 1 Introduction

Impacted ureteral calculi (IUC) are difficult to move because they are surrounded by edematous ureteral mucosa or polyps, accompanied by moderate hydronephrosis or stones staying in the same position for more than 2 months.^[1–3]^ IUC can cause kidney damage if the ureteral obstruction is not removed in time.^[1–4]^ Recent studies have suggested that the diagnosis and treatment of delayed ureteral obstruction is the most important prognostic factor for the poor outcome of renal function recovery, with the risk of hypertension or worsening of hypertension.^[5]^ IUC are often accompanied by hydronephrosis, infection, and other pathological changes, so clinical treatment is difficult.^[3–7]^ Currently, ureteroscopic holmium laser lithotripsy (UHLL) and ureteroscopic pneumatic lithotripsy (UPL) are the most commonly used methods in the treatment of IUC.^[8,9]^ However, there are still some controversies on the effect and complications of UHLL and UPL in the treatment of IUC. Some scholars believe that UPL uses mechanical energy lithotripsy, no thermal damage, and less damage to the ureter. However, when encountering stones with greater hardness, there are disadvantages such as difficulty in crushing stones, prolonged operation time, and migration of stones.^[10,11]^ Some scholars believe that UHLL has a relatively high efficiency of lithotripsy; however, there are shortcomings such as migration of some stones and thermal damage to the ureter, especially when the field of vision is not clear, the lithotripsy is more likely to produce ureteral injury.^[10,11]^ Therefore, we retrospectively analyzed the clinical data of 280 patients who underwent UHLL and UPL in our hospital from April 2016 to May 2019, and compared the effectiveness and safety of UHLL and UPL in the treatment of IUC.

## 2 Method and material

### 2.1 Case collection

The clinical data of 280 patients who underwent UHLL and UPL in our hospital from April 2016 to May 2019 were analyzed, including 136 cases in the UHLL group and 144 cases in the UPL group. Inclusion criteria: the patient was diagnosed as IUC by clinical consultation, urinary ultrasound, and urinary computed tomography (CT) examination; the patient was operated on for the first time. Exclusion criteria: those with urinary tract abnormalities; those with severe cardiopulmonary intolerance to surgery; those with immune system diseases; those with coagulopathy; people with severe hepatorenal dysfunction; people with a previous history of ureteroscopy; people with other organ infections and fever diseases. IUC refer to the retention of calculi in the same position of ureter for more than 2 months or the presence of moderate to severe hydronephrosis near the calculi.^[12,13]^ All patients included in the study signed the informed consent form with the patients themselves and their families, and the study was approved by the Ethics Committee of Yuechi people's Hospital.

### 2.2 Surgical methods and clinical data collection

The operation methods and standard procedures of UHLL and UPL are the same as those reported previously.^[12–15]^ All the operations in this study were performed by the same senior surgeon. After UHLL and UPL treatment, the stone fragments are taken out with a stone basket (Cook, Inc, IN). The holmium laser is made by American Lumenis Physician Medical Laser and matched optical fiber. The German wolf F8/9.8 ureteroscope was used. The pneumatic ballistic stone crusher and the matching handle needle of EMS company of Switzerland are used. The laser pulse energy is set as 0.8 to 1.5 J, and the frequency is 4 to 12 Hz. The double J ureteral tubes were retained for 30 days after the operation. The day after the operation, abdominal plain film and CT were used to evaluate the stone-free rate (SFR). If the image shows that the stone completely disappears or the residual stone in the urinary tract is less than 4 mm, it is regarded as stone removal. If the stone fragments are greater than or equal to 4 mm, the stones are considered residual. The stones were examined by noncontrast enhanced scan, and classified according to the position of the stones: proximal (above the iliac spine) and distal (below the iliac spine). The stone diameter is measured by noncontrast enhanced, and the maximum lateral diameter of the stone is recorded as the stone diameter. Clinical data such as general clinical treatment, operation time, hospital stay, hospitalization cost (mainly collect relevant expenses of patients on the day of operation, including operation cost, anesthesia cost, consumables cost, medicine cost, etc), SFR, intraoperative blood loss (intraoperative bleeding volume (ML) = hemoglobin concentration in flushing solution (g/L) ×flushing solution (L)/hemoglobin concentration of patients before operation (g/L) ×1000), and surgical complications were recorded.

### 2.3 Statistical processing

SPSS 19.0 software was used for data analysis. The continuous or categorical data are presented as mean ± standard deviation, frequency, percentile, and range, as appropriate. K to S single sample test was used to calculate the normal distribution of continuous variables before doing further comparison. Student *t* test and the Wilcoxon test were used to compare clinical characteristics between UHLL and UPL groups. Variables in the contingency table were analyzed by the χ^2^ test (or the Fisher exact test). When *P* < .05, the difference was significant.

## 3 Results

### 3.1 Comparison of general clinical data between the 2 groups

As shown in Table 1, the 2 groups were similar in gender (*P* = .437), age (*P* = .308), BMI (*P* = .615), stone diameter (*P* = .515), stone location (*P* = .102), stone size (*P* = .802), and comorbidities (*P* > .05) with no statistically significant difference. The results suggested that the demographic and stone characteristics of the 2 groups were similar.

**Table 1:**
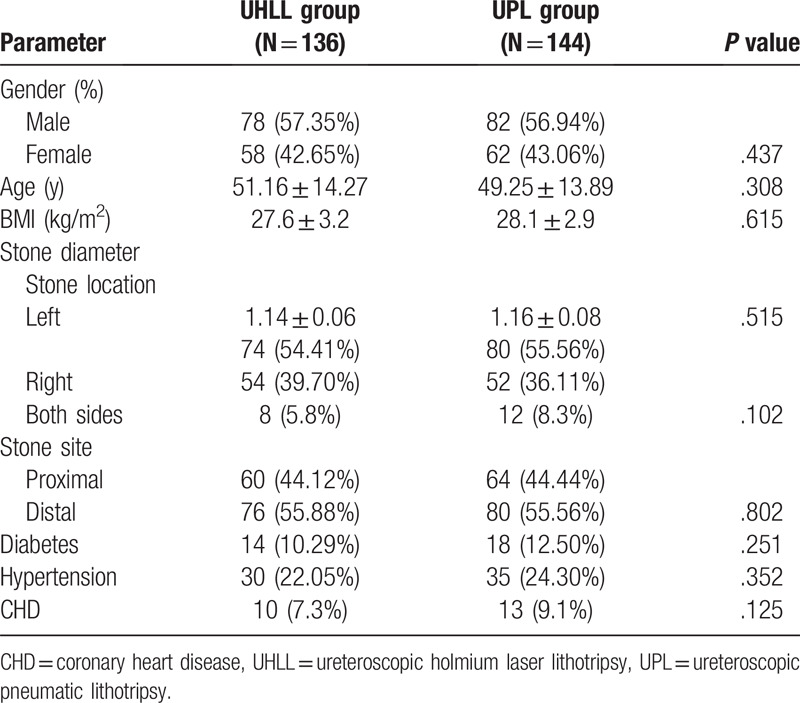
Comparison of clinical baseline data between the 2 groups.

### 3.2 Comparison of surgical index between the 2 groups

As shown in Table 2, there was no statistical difference between the 2 groups in terms of hospital stay and intraoperative bleeding. Compared with the UPL group, the operation time of UHLL group was significantly reduced (27.25 ± 8.39 vs 34.32 ± 10.57, *P* < .05), but the hospitalization cost was significantly increased (9.25 ± 0.75 vs 8.24 ± 0.51, *P* < .05), which suggested that UHLL could significantly shorten the operation time, but could not reduce the hospitalization cost of patients.

**Table 2:**
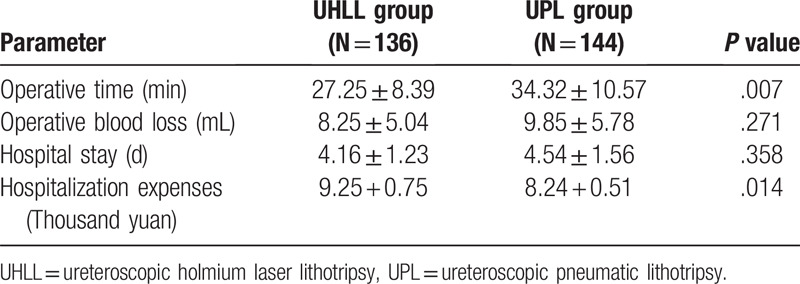
Comparison of surgical index between the 2 groups.

### 3.3 The comparison of the SFR of operation on different parts of IUC in 2 groups

**Figure 1:**
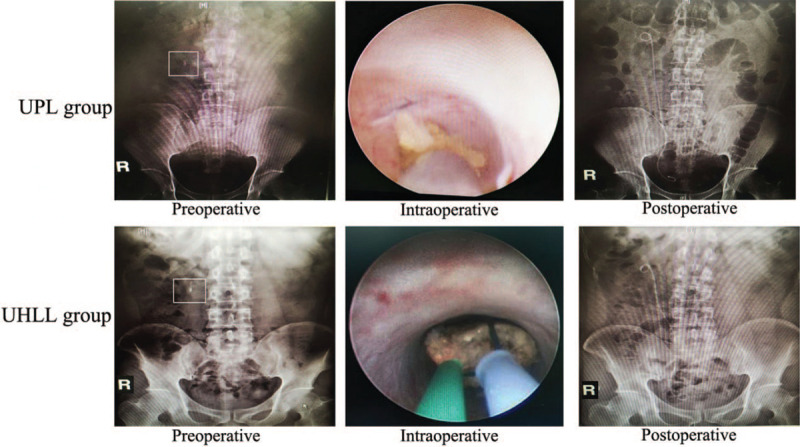
An example image of UHLL and UPL therapy for IUC before, after, and during operation.

Figure 1 is a typical picture of UHLL and UPL therapy for IUC before, after and during operation. As shown in Table 3, the UHLL group was significantly higher than the UPL group (93.38% vs 83.33%, *P* = .011) in terms of total SFR. For proximal IUC, compared with the UPL group, the SFR was significantly increased (88.33% vs 70.31%, *P* = .005). For distal IUC, there was no significant difference in SFR (97.37% vs 93.75%, *P* = 0.638) between the UHLL group and UPL group. These results suggest that UHLL is more effective than UPL in the removal of IUC.

**Table 3:**
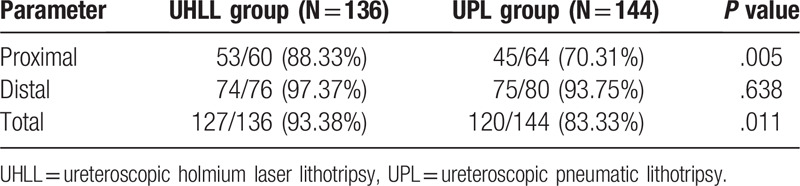
Comparison of the stone clearance rate of incarcerated ureteral calculi in different parts between the 2 groups.

### 3.4 Comparison of operative complications between the 2 groups

As shown in Table 4, patients in UHLL group and UPL group had no statistical significance in the complications of local mucosal injury (13.23% vs 14.58%, *P* = .585), gross hematuria (47.05% vs 52.77%, *P* = .145), fever urinary tract infection (9.27% vs 8.33%, *P* = .329), ureteral perforation (3.67% vs 5.55%, *P* = .105), urosepsis (3.67% vs 4.16%, *P* = .226). There were no serious complications such as avulsion of ureteral mucosa in UHLL group and UPL group. But, compared with the UPL group, the stone residual rate of UHLL group was significantly lower than that of UPL group (6.61% vs 16.67%, *P* = .001), which suggested that UHLL group had more advantages in the treatment of IUC.

**Table 4:**
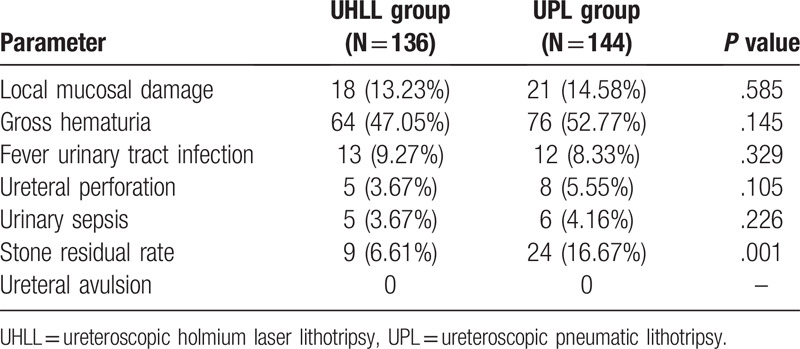
Comparison of surgical complications between the 2 groups.

## 4 Discussion

IUC have always been a difficult type of calculi in urology. Because IUC often adhere to ureteral mucosa or are wrapped by polyps, the traditional extracorporeal shock wave lithotripsy is difficult to work on them. In the past, the treatment of this kind of calculi mostly used open surgery.^[16]^ With the development of surgical techniques and instruments, minimally invasive techniques such as ureteroscopic lithotripsy and percutaneous nephrolithotripsy are widely used to treat IUC.^[17,18]^ But in all minimally invasive techniques, ureteroscopic lithotripsy is the minimally invasive technique with the least trauma and the fastest postoperative recovery.^[19,20]^ In ureteroscopic lithotripsy, the use of holmium laser and pneumatic ballistics significantly improved the stone removal efficiency. Holmium laser uses pulsed energy to pulverize ureteral stones. Holmium laser lithotripsy produces smaller fragments, even smaller than 1 mm in diameter. In addition, holmium laser lithotripsy has less damage to the ureter, less edema after ureteral operation, and smaller stone fragments can spontaneously flow out of the body with urine through the ureter.^[21,22]^ Pneumatic ballistic lithotripsy (PBL) is a cheap, safe, and effective lithotripsy technique, which can remove ureteral calculi in most patients. The energy of PBL is stable, and the price is lower than holmium laser. But PBL needs a broad and continuous working channel, and is easy to cause the stone to move forward, which is more obvious in the upper ureteral calculi.^[12,23]^

In our study, we found no significant difference in intraoperative blood loss and hospital stay between UHLL and UPL groups. Compared with the UPL, the operation time of UHLL group was significantly reduced (*P* < .05), but the hospitalization costs were significantly increased (*P* < .05), which suggested that UHLL could significantly shorten the operation time, but could not reduce the hospitalization costs of patients. The main reason for this result is that holmium laser energy is large, the blasting effect on stone is strong, the stone removal efficiency, and speed are fast, but the laser fiber belongs to high value consumables and the price is high. Previous studies have suggested ureteroscopic lithotripsy in the treatment of ureteral calculi SFR of 72% to 91%.^[24,25]^ In this study, we found that the SFR of UHLL group was about 93.38%, and that of UPL group was about 83.33%. The total SFR of UHLL group was significantly higher than that of UPL group (*P* < .05), which was similar to the previous literature.^[12–15,24,25]^ Although UHLL was more effective than UPL in the treatment of proximal IUC (88.33% vs 70.31%, *P* < .05). However, the efficiency of UHLL and UPL for distal impacted ureteral calculi is similar, and the difference is not statistically significant (*P* > .05).

Ureteroscopic lithotripsy can cause complications such as ureteral perforation, stone migration and sepsis, among which the most serious complication is ureteral avulsion.^[26–28]^ In this study, we found that there was no significant difference between UHLL group and UPL group in the incidence of local mucosal injury, postoperative hematuria, infection, ureteral perforation, urinary sepsis, and other complications (*P* > .05). In 280 cases of ureteroscopic lithotripsy, there was no serious complication of avulsion of ureteral mucosa, and the complications of ureteral perforation and uremia were also low. The complications of ureteral perforation, hematuria, and fever urinary tract infection were mild in some patients. We used conservative treatment, indwelling ureteral stent, antiinfection, and symptomatic treatment to cure them. However, in this study, we found that stone residual rate of UHLL group was significantly lower than that of UPL group (6.61% vs 16.67%, *P* = .001), which suggested that UHLL had more advantages in the treatment of IUC. Although this study found that the UHLL has more advantages in treating IUC, the study still has some limitations. Firstly, this study is a retrospective study and is not a double-blind randomized controlled trial in design. Secondly, the sample size of the study is small and cannot fully represent the actual situation in all cases. Thirdly, this study has all the limitations and risks of bias inherent in the study design. Fourthly, due to the differences in the operating experience of the operating personnel, the results of this study can only represent the research conclusions of the unit, and the department can be extended to different populations.

In conclusion, we found that both UHLL and UPL are safe and effective in the treatment of IUC, but UHLL has the advantages of short operation time and high stone removal efficiency in the treatment of IUC.

## Author contributions

**Conceptualization:** Yang Chunlin, Du Wanlin.

**Data curation:** Yang Chunlin, Du Jinhua.

**Formal analysis:** Yang Chunlin, Du Jinhua, Du Wanlin.

**Methodology:** Yang Chunlin, Du Jinhua, Du Wanlin.

**Writing – original draft:** Yang Chunlin, Du Jinhua, Du Wanlin.

**Writing – review & editing:** Yang Chunlin, Du Jinhua, Du Wanlin.
